# Clinical presentation, treatment and outcome of paraphenylene-diamine induced acute kidney injury following hair dye poisoning: a cohort study

**DOI:** 10.11604/pamj.2014.19.163.3740

**Published:** 2014-10-16

**Authors:** Mazin Shigidi, Osama Mohammed, Mohammed Ibrahim, Elshafie Taha

**Affiliations:** 1 Faculty of Medicine, University of Khartoum, Sudan; 2 Renal Unit, Khartoum Teaching Hospital, Sudan; 3 Cardiology Department, Al Ain Hospital, United Arab Emirates

**Keywords:** Hair dye poisoning, paraphenylene-diamine, acute kidney injury

## Abstract

**Introduction:**

In Africa and Asia hair dye is applied together with henna to decorate the hands and feet. Paraphenylene-diamine (PPD), a highly toxic constituent of hair dye can lead to acute kidney injury (AKI).

**Methods:**

A cohort study was conducted during the period from April 2012 to March 2013 in Khartoum Teaching Hospital, Sudan. It targeted adults presenting acutely with an evident history and clinical features of hair dye poisoning, together with AKI as per the RIFLE criteria. Analysis of data was done using SPSS.

**Results:**

30 adults were included, their mean age was 25.6 ± 4.2 years, 93.3% were females. Exposure to PPD was suicidal in 86.7%. The mean duration to onset of renal symptoms was 34.8 ± 7.6 hours, maximum median serum creatinine was 8.6 ± 2.3 mg/dl, 86.7% had loss of kidney function as per the RIFLE classification and required dialysis. Initial renal recovery was seen after a mean duration of 9.8 ± 2.2 days. One patient died, 3.3%; all others, 96.7%, recovered normal kidney function. The amount of ingested PPD correlated significantly to the severity of symptoms, number of dialysis sessions required and time for renal recovery with P values < 0.05.

**Conclusion:**

Hair dye poisoning was associated with prolonged hospital stay, requirement of dialysis and increased morbidity. The severity of symptoms directly correlates to the dose of PPD ingested, with the kidney damage being reversible in almost all survivors.

## Introduction

In Africa and Asia hair dye is applied by women as a cosmetic agent together with henna, leaves of a non-toxic herb (*Lawsonia inermis*). The combination, Black henna, is applied as a temporary tattoo to decorate the hands and feet in special social events such as wedding ceremonies [1, 2]. Paraphenylene-diamine (PPD) is a main constituent of hair dye formulations and known to be highly toxic [2–4]. Variable concentrations of PPD are used in different hair dye formulations with the exact concentration that cause toxicity being undetermined [5]. Systemic intoxication with PPD commonly occur via the oral and trans-dermal routes [4]. Hair dye poisoning had been widely reported in Sudan, with 3 159 cases being described during the period between 1995 and 2005 [6–8]. It had been associated with increased patients'morbidity and mortality [1, 9]. Acute kidney injury (AKI) following hair dye poisoning and exposure to PPD is multifactorial, with the Kidney damage being mostly due to a direct toxic effect of PPD, hypovolemia, hemolysis and rhabdomyolysis [10]. This study aimed to determine the clinical presentation, management and outcome of PPD induced AKI, following hair dye poisoning, among Sudanese adults.

## Methods

A prospective hospital-based cohort study was conducted in Khartoum Teaching Hospital, Khartoum, Sudan. It included all adult patients presented acutely to the Emergency Department and labeled as having PPD induced AKI, following hair dye poisoning. Paraphenylene-diamine induced AKI was considered evident by a definitive history of exposure to hair dye together with the clinical manifestations of PPD intoxication [11]. The presence of AKI was defined and graded as per the RIFLE criteria [12, 13].

Children, patients with chronic kidney disease, those with prior history of exposure to PPD or other nephrotoxins three months before presentation, patients with acute or chronic liver disease, and those who refused to give consent for enrollment were all excluded from the study. Patients'history, clinical examination, laboratory investigations, management and follow-up plan were observed from the time of hospital admission till discharge or death. The amount of hair dye ingested was estimated by direct questioning. Patients were provided hemodialysis on per need basis if they developed symptoms of uremia, serious complications of uremia, pulmonary edema not responding to diuretics, severe metabolic acidosis, hyperkalemia not controlled by conservative measures and rising blood urea with levels more than 200 mg/dl [13, 14]. Onset of renal recovery was determined once the patient is non-oligoanuric, together with improving serum creatinine despite withdrawal of dialysis. On the other hand, Patients were labeled as recovered kidney function once serum creatinine levels are back to within normal reference range (0.6 to 1.5 mg/dl) despite being off dialysis therapy [13]. Data collection, patients'assessment, treatment and follow-up were all done by a single physician. Again all investigations were done in a single laboratory using a standardized technique.

Data obtained were entered into a computer software via a specially designed questionnaire. Analysis of data was done using Statistical Package for the Social Sciences version 17.0 (SPSS, Inc., Chicago, IL, USA). Descriptive analysis was done for all variables. Variables were expressed as percentages, means and / or medians with standard deviations. Comparison of data was done using the Student′s t test for continuous variables and Fisher′s exact X^2^ test for categorical variables with levels of significance being set at P values of less than 0.05. Ethical clearance was obtained from Sudan Medical Specialization Board. Consent for enrollment was obtained from all patients.

## Results

During the period from April 2012 to March 2013 a total of 30 adult Sudanese patients were admitted to Khartoum Teaching Hospital Emergency Department with hair dye poisoning and PPD induced AKI as per the inclusion / exclusion criteria set. All patients were using the same commercial brand of hair dye. The mean age of the study population was 25.6 ± 4.2 years, the majority were females, 93.3%, two-third of patients were having a secondary high school education level, 60% were unmarried, 43.3% unemployed and 26.7% were housewives. Exposure to PPD was via the oral route in all patients, 100%. Reasons for ingestion were a suicidal attempt in 86.7%, accidental in 10% and a suspected homicide in 3.3% (Table 1).

**Table 1:** Demographic features of the study population

Demography	Frequency (%)
Total number of patients studied	30 (100%)
Female / Male ratio	28 (93.3%) / 2 (6.7%)
Married / Unmarried	12 (40%) / 18 (60%)
**Age groups**	
Less than 20 years	2 (6.7%)
20 - 30 years	15 (50%)
30 - 40 years	11 (36.7%)
More than 40 years	2 (6.7%)
**Residency**	
Urban	27 (90%)
Rural	3 (10%)
**Employment**	
Office work	5 (16.7%)
Student	4 (13.3%)
Housewife	8 (26.7%)
Unemployed	13 (43.3%)
**Education**	
Graduate	4 (13.3%)
High school	23 (76.7%)
Primary school	2 (6.7%)
None	1 (3.3%)
**Past history of suicidal attempts**	2 (6.7%)

The mean duration from time of exposure to the appearance of renal symptoms was 34.8 ± 7.6 hours; these were mostly in the form of reduced urine output and the passage of dark chocolate brown urine seen in 90% and 76.7% of patients, respectively. Angioneurotic edema was seen in 53.3% of patients with emergency tracheostomy being done in all of them. All patients had normal echocardiographic studies despite that cardiac arrhythmias were documented in 40%.

As per the AKI RIFLE classification most patients, 86.7%, had loss of kidney function and required dialysis therapy. In 73.3% of patients serum creatinine peaked to more than 10 mg/dl, with the maximum median serum creatinine for all patients being 8.6 ± 2.3 mg/dl. Dialysis replacement therapy was indicated in 86.7%, with only 13.3% of patients being treated conservatively. Indications for dialysis were mostly oliguria with rising blood urea of more than 200 mg/dl, uremic encephalopathy and pulmonary edema in 63.3%, 13.3% and 10% of patients, respectively. The average number of hemodialysis sessions required was 5 ± 1 sessions. Intradialytic complications in the form of hypotension and hypoglycemia were evident in 10%. Initial signs of renal recovery were seen after a mean duration of 9.8 ± 2.2 days. Overall, 96.7% of patients recovered normal kidney function, whereas 3.3% died. None of the study patients developed end-stage renal disease (Table 2).

**Table 2:** Clinical features of hair dye poisoning among the study population

Clinical features	Frequency (%)
**Amount of PPD ingested**	
5 - 10 gm	4 (13.3%)
10 - 15 gm	24 (80%)
More than 15 gm	2 (6.7%)
**Time from PPD exposure to symptoms**	
Less than 24 hours	3 (10%)
24 - 36 hours	11 (36.7%)
36 - 48 hours	9 (30%)
More than 48 hours	7 (23.3%)
**Clinical manifestations**	
Neurological manifestations	3 (10%)
Gastrointestinal symptoms	5 (16.7%)
Chest symptoms	8 (26.7%)
Arrhythmias	12 (40%)
Angioneurotic edema	16 (53.3%)
Change in urine color	23 (76.7%)
Oliguria	27 (90%)
Acute kidney injury	30 (100%)
**AKI RIFLE Classification**	
Risk	0 (0%)
Injury	0 (0%)
Failure	4 (13.3%)
Loss of Function	26 (86.7%)
End-stage Renal Disease	0 (0%)
**Median maximum serum creatinine**	8.6 ± 2.3 mg/dl
**Patients required dialysis / No dialysis**	26 (86.7%) / 4 (13.3%)
**Mean number of dialysis sessions**	5 ± 1 sessions
**Patients’ outcome**	
Recovered	29 (96.7%)
Died	1 (3.3%)
**Mean duration of hospital stay**	14.6 ± 3.4 days

Among the study group the dose of PPD ingested correlated significantly to the severity of symptoms in the form of occurrence of pulmonary edema, cardiac arrhythmias, high serum creatinine levels at presentation, requirement of dialysis therapy, number of dialysis sessions required and duration to onset of renal recovery; P values being less than 0.05 (Table 3). Furthermore, the presence of pulmonary edema at presentation, cardiac arrhythmias and level of serum creatinine were all found to be significant predictors when correlated to the time for renal recovery, with P values of less than 0.05 (Table 4).

**Table 3:** Amount of hair dye ingested and its impact on patients’ presentation and outcome

Clinical Manifestations	Ingested amount of hair dye			P value
	5-10 gm N = 4	10-15 gm N = 24	> 15 gm N = 2	
**Occurrence of pulmonary edema**	0 (0%)	6 (25%)	2 (100%)	0.03
**Serum creatinine at presentation**				0.01
1.5 - 5 mg/dl	1 (25%)	0 (0%)	0 (0%)	
5 - 7.5 mg/dl	2 (50%)	1 (4%)	0 (0%)	
7.5 - 10 mg/dl	0 (0%)	4 (17%)	0 (0%)	
More than 10 mg/dl	1 (25%)	19 (79%)	2 (100%)	
**Required dialysis**	1 (25%)	23 (96%)	2 (100%)	0.0001
**Number of hemodialysis sessions**				0.016
None	3 (75%)	1 (4%)	0 (0%)	
1 - 3 sessions	0 (0%)	1 (4%)	0 (0%)	
3 - 5 sessions	0 (0%)	9 (38%)	1 (50%)	
More than 5 sessions	1 (25%)	13 (54%)	1 (50%)	
*Onset of renal recovery				0.0001
Less than 1 week	3 (75%)	0 (0%)	0 (0%)	
1 - 2 weeks	1 (25%)	17 (71%)	1 (50%)	
More than 2 weeks	0 (0%)	6 (25%)	1 (50%)	

* Only 29 out of 30 patients showed renal recovery as one patient died

**Table 4:** Predictors of delayed renal recovery following hair dye poisoning

Clinical Manifestations	Onset of renal recovery			P value
	< 7 days N = 3	7-14 days N = 20	> 14 days N = 6	
**Presence of pulmonary edema**	0 (0%)	4 (20%)	4 (66.7%)	0.04
**Presence of cardiac arrhythmias**	0 (0%)	5 (25%)	6 (100%)	0.001
**Serum creatinine at presentation**				0.001
1.5 - 5 mg/dl	1 (33.3%)	0 (0%)	0 (0%)	
5 - 7.5 mg/dl	2 (66.7%)	1 (5%)	0 (0%)	
7.5 - 10 mg/dl	0 (0%)	3 (15%)	1 (1.7%)	
More than 10 mg/dl	0 (0%)	16 (80%)	5 (83.3%)	

Total number of patients recovered 29

## Discussion

Hair dye poisoning had been repeatedly reported as an important cause of suicide and para-suicide, with various case series being described from East Africa, the Middle East and the Indian subcontinent [5, 7, 10, 15–19]. In our study the young unmarried females were the dominant patients, a finding consistent with prior published reports from Sudan [4, 8].

Hair dyes contain PPD at various concentrations ranging from 0.2% to 3.75%. It is PPD, the toxic ingredient causing laryngeal edema, rhabdomyolysis, severe metabolic acidosis and AKI following hair dye poisoning [20]. All patients included in the study were exposed to PPD through the oral route. In two-third of patients the symptoms of toxicity were evident within 48 hours after exposure. In the literature, clinical features of toxicity had occasionally been reported via trans-dermal absorption as well [8]. Few days after exposure, patients tend to pass chocolate-brown colored urine, develop rhabdomyolysis and ultimately AKI. The reported incidence of AKI following hair dye poisoning range from 47.4% to 90%, [8, 9] with rhabdomyolysis, hemolysis, the presence of hypovolemia as well as the direct toxic effect of PPD on the renal tubular cells being the cause; histological evidence of acute tubular necrosis had been repeatedly observed [3, 9, 21].

Renal involvement post hair dye poisoning can be transient and pass unnoticed; again it can be severe enough to necessitate dialysis [9, 21]. In one report from the Sudan, dialysis replacement therapy was required in 60% of cases with PPD induced AKI, with an average of 5 dialysis sessions being provided before renal recovery is achieved [8, 22]. Despite its limitations, serum creatinine remains the most commonly used indicator of kidney damage. Its high levels had been strongly linked to poorer clinical outcomes [23]. In our study serum creatinine was monitored to determine renal recovery; with the higher levels at presentation being associated with delayed renal recovery and a longer hospital stay [24, 25]. Among our patients 86.7% were having severe kidney damage necessitating dialysis replacement therapy with almost all patients showed full renal recovery [4, 8, 23]. The PPD toxin is not known to be dialyzable; there is no specific antidote for hair dye poisoning and treatment is mainly supportive [11]. Reported patients'mortality following poisoning range from 0.03% to 60%, with the commonest causes of death being angioneurotic edema and cardiac arrhythmias; our case fatality was 3.3% [3, 20].

## Conclusion

Hair dye poisoning had been widely reported in Sudan with the majority of patients being young females. Most of those who develop developed AKI require dialysis. The condition is associated with multisystem involvement, prolonged hospital stay, requirement of dialysis therapy, increased morbidity and a high risk of death due to angioneurotic edema and cardiac arrhythmias. The severity of symptoms was directly related to the dose of PPD ingested.
